# Conjugated Hyperbilirubinemia in Infants: Is There Still a Role for ERCP?

**DOI:** 10.1155/2021/9969825

**Published:** 2021-06-24

**Authors:** Jan Stovicek, Stepan Hlava, Radan Keil, Jiri Drabek, Jindra Lochmannova, Petra Koptová, Martin Wasserbauer, Barbora Frybova, Jiri Snajdauf, Radana Kotalova, Michal Rygl

**Affiliations:** ^1^Department of Internal Medicine, Charles University in Prague, 2nd Faculty of Medicine, University Hospital Motol in Prague, Prague, Czech Republic; ^2^Department of Pediatric Surgery, Charles University in Prague, 2nd Faculty of Medicine, University Hospital Motol in Prague, Prague, Czech Republic; ^3^Department of Pediatry, Charles University in Prague, 2nd Faculty of Medicine, University Hospital Motol in Prague, Prague, Czech Republic

## Abstract

Over a twenty-year period, we performed 255 ERCP procedures in infants aged up to 1 year. ERCP was indicated in cholestatic infants with suspicion of biliary obstruction. The most common diagnosis was biliary atresia (48%), choledochal cysts (13%), and choledocholithiasis (4%). The procedure complication rate was 13.7%. Hyperamylasemia occurred in 12.9%. More severe complications were rare‐0.8% of ERCP procedure. There were no cases of postprocedural pancreatitis or death. Our study has proved that ERCP is a safe and reliable method in this age group. Its high specificity and negative predictive value for extrahepatic biliary atresia can prevent unnecessary surgeries in patients with normal bile ducts or endoscopically treatable pathologies.

## 1. Introduction

Cholestasis in children under one year is a serious condition with multiple etiologies. ERCP is one of the diagnostic and potentially therapeutic methods that can distinguish between a surgical and a nonsurgical etiology of cholestasis.

Indications for ERCP differ in various age groups. In the group of children aged less than 1 year, ERCP is a useful method of confirming or excluding biliary atresia and pancreaticobiliary maljunction.

Newborns are indicated mainly for neonatal cholestasis with the goal to exclude or confirm biliary atresia. The unnecessary surgery can be avoided if ERCP fading confirms a normal biliary tract.

The second most frequent indication is suspicion of a choledochal cyst in patients with obstructive jaundice. Typically in these cases, there is a relatively small dilatation of the bile duct, with plugs stemming from protein debris from the wall of the bile duct. Insertion of a biliary stent can postpone the necessity of surgery at an older age.

The aim of this study is to determine the safety of the method and demonstrate its indispensable position/role in the diagnostic algorithm.

## 2. Patients and Methods

ERCP procedures performed in cholestatic infants aged 1 year or younger, performed from January 2000 till December 2020, were analyzed retrospectively.

**Figure 1:**
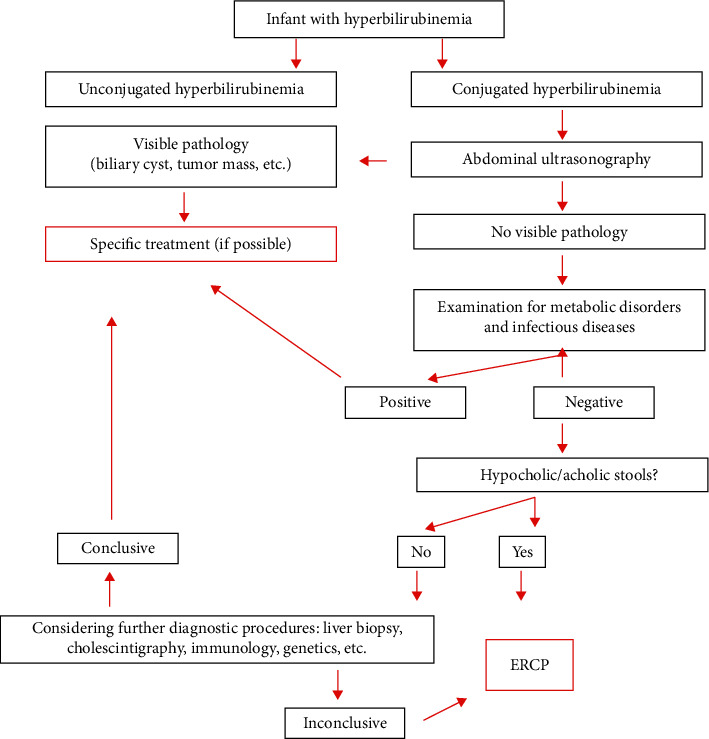
Diagnostic algorithm for conjugated hyperbilirubinemia.

ERCP was indicated in a subgroup of cholestatic infants with a suspicion of extrahepatic biliary obstruction. The standardized algorithm of diagnostic workup adopted at our institution was used (Figure 1).

Three outcomes were evaluated: the rate of technical success, the correlation of ERCP findings with the final diagnosis, and the rate of complications. Patients were divided into subgroups according to their diagnosis, and these subgroups were analyzed in more detail, including the subgroup of patients with a procedural technical failure. The data were statistically analyzed: sensitivity, specificity, and positive (PPV) and negative (NPV) predictive values were calculated for biliary atresia.

All ERCP procedures were performed by two experienced endoscopists. The examinations were carried out on a fluoroscopic table under general anesthesia, with continuous monitoring of vital functions. In all cases, the pediatric duodenoscope (Olympus PJF) with a 7.5 mm outer diameter and 2 mm working channel was used. The lowest body weight in our cohort was 1.4 kg. Ultrathin handmade cannulas were used for cannulation. For the insertion of stents 5 Fr in diameter, it was necessary to use a double-lumen sphincterotome with an outer diameter of 1.75 mm (Medi-Globe RotaCut GSP-21-17-020) and a guide wire with an outer diameter of 0.53 mm (Cook METII-21-480 Tracer Metro Direct Wire Guide). The therapeutic role of ERCP was limited by the fact that, until 2018, sphincterotomy was not performed with the pediatric duodenoscope as the manufacturing company did not recommend it.

In patients with biliary atresia, the findings were divided according to Guelrud's classification [1]: type 1: no visualization of the biliary tree, type 2: opacification of the distal common duct and the gallbladder without visualization of the main hepatic duct, and type 3: opacification of the distal common duct, the gallbladder, and a segment of the main hepatic duct with biliary lakes at the porta hepatis.

**Figure 2:**
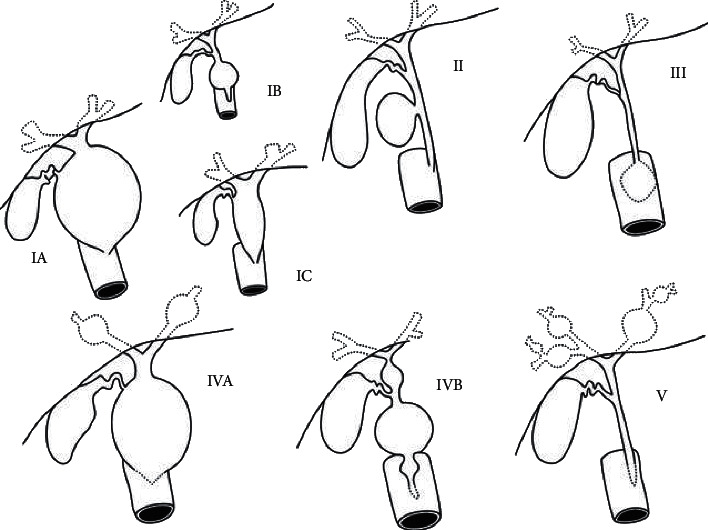
Todani classification of biliary cysts.

Todani classification was used to evaluate biliary cyst findings: type IA: a cystic dilatation of the extrahepatic biliary tree, type IB: a focal, segmental dilatation of the extrahepatic bile duct, type IC: a smooth fusiform dilatation of the entire extrahepatic bile duct, type II: a discrete diverticula of the extrahepatic duct, type III: choledochocoele, type IVA: a combination of intrahepatic and extrahepatic duct dilatation, type IVB: multiple extrahepatic bile duct dilatation, and type V: Caroli disease [2] (Figure 2). The findings of anomalous pancreaticobiliary junction (common channel) were documented, but not further classified.

The retrospective analysis was approved by the Ethics Committee of the University Hospital Motol and 2nd Faculty of Medicine, Charles University in Prague (reference no. EK-1100/18).

## 3. Results

ERCP procedures were performed on 255 infants (113 girls and 142 boys) aged 1 year and younger between January 2000 and December 2020. The average age of the patients at the time of procedure was 12.1 weeks. The infants were indicated for ERCP for conjugated hyperbilirubinemia and laboratory signs of cholestasis.

The dominating finding was biliary atresia (BA) (121 children, 48% of all procedures), followed by choledochal cyst (34 children, 13% of all procedures) and choledocholithiasis (9 children, 4% of all procedures) (Table 1). Other findings were marginal.

66 patients (26% of all procedures) had a normal finding.

Biliary atresia was diagnosed in 121 infants. The mean age of these patients at the time of procedure was 8.6 weeks, median: 7.7 weeks. The predominant finding was biliary atresia type I (99 infants, 82%), and biliary atresia type II was found in 22 patients (18%).

**Figure 3:**
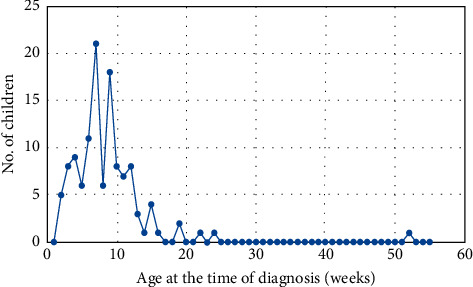
Age distribution of infants with biliary atresia at the time of diagnosis.

The age distribution of children with biliary atresia is shown in Figure 3.

False positive diagnosis of BA was established in 10 patients (8.3%)—5 had cholestasis only, 3 had Alagille syndrome, 1 had bile duct obstruction, and 1 had bile duct hypoplasia. Surgical revision was performed only in 4 of them. Two patients with Alagille syndrome underwent liver transplantation, and 1 infant with Alagille syndrome died before transplantation.

The positive predictive value for BA is 91.8%, the negative predictive value is 100%, specificity is 93.1%, and sensitivity is 100%.

Biliary cyst was diagnosed in 34 kids (13% of all procedures). The mean age of the patients with the biliary cyst finding was 15.9 weeks, median: 10 weeks. Frequency of cyst types according to Todani classification is shown in Table 2.

ERCP procedures failed in 12 patients due to technical reasons. The reasons (for technical failure) were duodenal stenosis (4 patients), a very small or an atypical papilla in an atypical localization (3 patients), a papilla was not found (4 patients), and situs organum viscerum (1 patient).

The overall complication rate was 13.7% of ERCP procedures. Asymptomatic hyperamylasemia occurred in 12.9%. It is questionable if asymptomatic hyperamylasemia should be included in the complication rate. The suspicion of perforation (abdominal pain and elevation of CRP) occurred in one cholestatic infant with a choledochal cyst after an unsuccessful biliary stent insertion. The problem was resolved conservatively with parenteral nutrition and intravenous antibiotic therapy. In one case, a retroperitoneal depot of the contrast medium emerged during the procedure. It was resolved conservatively (parenteral nutrition and antibiotic therapy). There were no cases of ERCP-induced pancreatitis. No mortality was observed after ERCP.

## 4. Discussion

Fast and correct diagnosis is crucial in the management of infants, mainly neonates, with cholestatic liver disease. The principal moment is most important to exclude extrahepatic biliary atresia in cholestatic infants. In spite of relative invasiveness, ERCP can be a very useful tool to reach this goal with sufficient specificity and sensitivity and a low severe complication rate. High specificity and the negative predictive value of ERCP for extrahepatic biliary atresia indicate a possibility to prevent surgery in patients with normal bile ducts or endoscopically treatable pathology. On the contrary, all papers promoting ERCP for this indication stress the importance of ERCP being performed in large-volume centers by experienced endoscopists.

Historically, the method of choice and the gold standard for the final diagnosis of bile duct atresia were intraoperative cholangiography that definitively demonstrates the anatomy and the patency of the extrahepatic biliary tract. It is recommended to perform intraoperative cholangiography when the liver biopsy findings suggest an obstructive etiology. The cholangiography is also indicated when biopsy results are equivocal or scintiscan fails to demonstrate clear evidence of duodenal bile excretion [3]. This method is more invasive and riskier for infants in comparison with ERCP.

Less-invasive methods used for this indication are no less problematic. Ultrasonography is noninvasive, cheap, and definitely useful to identify anatomic abnormalities, but reported sensitivity as low as 74.9% and specificity at 93.4% [4] were found unreliable in the evaluation of biliary atresia [3]. Triangular cord sign is supposed to be specific for biliary atresia, but its diagnostic usefulness is diminished by its technical difficulty and variability of interpretation of ultrasonographists. Cholescintigraphy possesses high sensitivity in biliary atresia diagnosis, but with low specificity (sensitivity: 93.4% and specificity: 69.2%) [3, 4].

Magnetic resonance cholangiopancreatography (MRCP) requires general anesthesia, even longer compared with ERCP. Technical advancement and clinical experience are necessary before it can be used in the evaluation of cholestatic infants. Sensitivity of MRCP is 89.7%, and specificity is only 64.7% [4]. It is not recommended to use MRCP as a routine test method alone [3].

Meta-analysis of noninvasive diagnostic methods shows growing specificity, and especially, a combination of different methods (ultrasound with MRI or ultrasound with hepatobiliary scintigraphy) might be the future of noninvasive diagnostics [4], but there are still no satisfying data to confirm this hypothesis. None of these methods has a 100% negative predictive value, which is extremely important for the treatment decision.

Diagnosis and treatment in infants aged up to 45 days are important for the best results of hepatic portoenterostomy. The results will progressively get worse if performed at the age of 60–90 days [5]. All patients with biliary atresia in our cohort were diagnosed prior to week 24, ERCP was performed, and diagnosis was established in average at the age of 9 weeks with median 8 weeks, with a peak of cumulation of cases in week 7 (Figure 3). The use of an appropriate and generally accepted diagnostic algorithm for diagnoses can be helpful. There may be some room for improvement in the future. Screening programs for acholic stool exist in some countries. For example, in Taiwan, stool color card screening reduced the age of atresia diagnosis significantly [6]. Stool cards have 76.5% sensitivity and 99.9% specificity for identifying children with biliary atresia [7]. Also, smartphone applications have been developed for this purpose [8].

**Figure 4:**
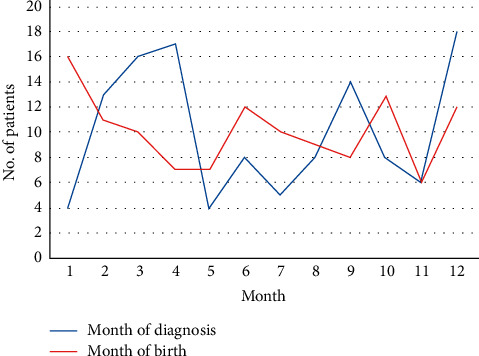
Number of biliary atresia by the month of birth and diagnosis.

Some studies have reported the seasonal variation of biliary atresia cases, suggesting a role of viral infections in the etiology of biliary atresia [9]. We looked at the distribution of the month of birth and the month of diagnosis throughout a year. We did not find any significant and repeating occurrence (Figure 4).

Biliary cysts were found in 12.9% of children, which is consistent with our previously published data. Biliary cyst or pathology of the pancreatobiliary junction was found in 10% of children examined by ERCP for cholestasis [10, 11]. ERCP in biliary cyst diagnosis offers therapeutic possibilities—sphincterotomy or stent insertion. Surgical treatment can thus be postponed to older age when surgery is safer and has better results [12]. The bile duct in infants is very narrow and fusiform like. On the contrary, biliary cysts in the infant age look usually just like a relative widening of the bile duct. This finding can be easily confused and described as normal.

Frequency of symptomatic choledochithiasis in patients younger than one year in this cohort is lower than that reported in our older publications, but still relatively high (3.5% versus 7.4%) [9]. The exact epidemiologic data of choledocholithiasis incidence in neonates and infants are missing, but it can be expected to be far less than 1 in 5000 [13], and in most cases, it is asymptomatic. Patients with symptomatic choledocholithiasis benefit from ERCP availability, and their length of stay in hospitals is shorter than without ERCP therapy [14].

Only 25% of patients had normal bile ducts with no pathology. It indicates a good preselection of patients who were referred to ERCP.

Despite all the positive features described above, the availability of ERCP for infants under one year will probably further decline because of the fact that the production of the pediatric duodenoscope (Olympus PJF) was discontinued in 2013. Several centers no longer have an infant ERCP duodenoscope due to breakdowns and wear [15]. If the production of these endoscopes is not restored, the diagnostic and therapeutic role of ERCP in neonates and infants will be endangered [16].

## 5. Conclusion

ERCP is a reliable and safe diagnostic method in children younger than one year if it is performed by an experienced endoscopist. It has an indispensable role in the diagnostic algorithm of cholestatic infants. Although specificity and sensitivity of combined noninvasive diagnostic methods are high, ERCP is a unique nonoperative method with 100% negative predictive value for biliary atresia diagnosis. Unfortunately, the termination of the production of pediatric duodenoscopes can lead to lower availability of this procedure in neonates and infants.

## Figures and Tables

**Table 1:** ERCP findings: types and frequency.

ERCP finding	Female	Male	Total
Biliary atresia	61	60	121
Type 1	55	44	99
Type 2	6	16	22
Bile cyst	10	24	34
Lithiasis	5	4	9
Stenosis	2	1	3
Pancreatic pathology	1	0	1
PSC	4	1	5
Postoperative pathology	3	2	5
Normal	19	47	66
Failed	8	4	12
Total	113	142	255

**Table 2:** Frequency of types of the cyst according to Todani classification.

Type of the cyst	No. of patients
Type 1A	7
Type 1B	13
Type IC	12
Type V	2
Total	34

## Data Availability

The data used to support the findings of this study are available from the corresponding author upon request.
